# Is malaria immunity a possible protection against severe symptoms and outcomes of COVID-19?

**DOI:** 10.4314/gmj.v55i2s.9

**Published:** 2021-06

**Authors:** Verner N Orish, Emily Boakye-Yiadom, Evelyn K Ansah, Robert K Alhassan, Kwabena Duedu, Yaw A Awuku, Seth Owusu-Agyei, John O Gyapong

**Affiliations:** 1 Department of Microbiology and Immunology, School of Medicine, University of Health and Allied Sciences, Ho, Volta Region, Ghana; 2 Centre for Malaria Research, Institute for Health Research, University of Health and Allied Sciences, Ho, Volta Region, Ghana; 3 Institute of Health Research, University of Health and Allied Sciences, Ho, Volta Region, Ghana; 4 Department of Biomedical Sciences, School of Basic and Biomedical Sciences, University of Health and Allied Sciences, Ho, Volta Region, Ghana; 5 Department of Internal Medicine, School of Medicine, University of Health and Allied Sciences, Ho, Volta Region, Ghana

**Keywords:** Malaria immunity, COVID-19, Innate immunity, Natural killer cells, interferon

## Abstract

Malaria-endemic areas of the world are noted for high morbidity and mortality from malaria. Also noted in these areas is the majority of persons in the population having acquired malaria immunity. Though this acquired malaria immunity does not prevent infection, it resists the multiplication of *Plasmodium* parasites, restricting disease to merely uncomplicated cases or asymptomatic infections. Does this acquired malaria immunity in endemic areas protect against other diseases, especially outbreak diseases like COVID-19? Does malaria activation of innate immunity resulting in trained or tolerance immunity contribute to protection against COVID-19? In an attempt to answer these questions, this review highlights the components of malaria and viral immunity and explores possible links with immunity against COVID-19. With malaria-endemic areas of the world having a fair share of cases of COVID-19, it is important to direct research in this area to evaluate and harness any benefits of acquired malaria immunity to help mitigate the effects of COVID-19 and any possible future outbreaks.

**Funding:**

None declared

## Introduction

Malaria, caused by the *Plasmodium* parasite, is an endemic disease found in most tropical countries, where environmental and socioeconomic factors foster its perennial persistence.^1^ In sub-Saharan Africa, malaria is a serious public health concern because the region bears a disproportionately high burden of global malaria morbidity and mortality, responsible for about 93% of the 228 million cases and 94% of 411,000 deaths globally in 2018.^1^ While everyone living in these endemic areas is at risk of falling ill or dying from malaria. Its impact is seen more among the vulnerable population, such as pregnant women and young children.^1^

Despite the high burden of malaria in these endemic areas, there is some level of malaria immunity among most of the population, acquired through a precarious, repeated lifelong encounter with the *Plasmodium* parasite.^2^,^3^ This malaria immunity is seen mainly among the population's non-pregnant adults, resulting in the mild or asymptomatic presentation of the disease among this group^2^, thus shifting the bulk of the morbidity and mortality to children and pregnant women.^1^ Apart from the acquired immunity from malaria actively preventing the severe outcomes of malaria, it is pertinent to ask if this immunity observed among the vast majority of the population living in malaria-endemic areas could also offer protection against other infectious diseases, such as COVID-19.

COVID-19 is a viral disease caused by a novel type of coronavirus called severe acute respiratory syndrome coronavirus 2 (SARS-CoV-2).^4^ It is an ongoing pandemic affecting all countries globally, including malariaendemic countries in sub-Saharan Africa.^5^ The global figures for COVID-19 as of December 17th 2020, were over 74 million with over 1.5 million deaths and over 42 million recoveries. For Africa was over two million with over 57 thousand deaths and over 2 million recoveries.^6^

There are several schools of thought for the relatively lower number of cases recorded in Africa, including inadequate testing of suspected cases and the prevailing warmer climate conditions.^7^,^8^ To date, no country within the malaria-endemic zone of Africa (i.e. West, Central and East Africa) has experienced sporadic spikes in mortality as observed in Europe, the Americas and the northern and southern parts of Africa. Could immunity to malaria, which is a predominant feature in these endemic areas, contribute to the low numbers of infections, faster clearance of the virus, milder presentation and low mortality observed so far? This brief review seeks to assess the possible role of malaria immunity in mitigating the impact of COVID-19 in malaria-endemic areas. This paper reviews the mechanisms of immune response to malaria and the dynamics of developing malaria immunity. We additionally review the basics of developing immunity against viruses, especially the coronaviruses and examine the dynamics of *Plasmodium* and viral coinfections, highlighting a possible relationship between acquired malaria immunity and coronavirus immunity.

### Developing Malaria Immunity

Malaria immunity is a complex phenomenon, an evolving topic yet to be fully understood.^2^,^9^ It is said to be a state of resistance to the *Plasmodium* parasite acquired through the repeated activities involving the clearance and restriction of the multiplication of the parasite.^9^ This acquired immunity begins as clinical immunity or antidisease immunity and effectively prevents the manifestation of symptoms within a given threshold of parasitemia.^2^,^9^,^10^ This means that, beyond a given threshold of parasite load, clinical immunity will fail.^9^ This type of acquired malaria immunity is first developed in childhood.^9^,^11^ The other type of acquired malaria immunity is the anti-parasite immunity responsible for direct parasite clearance with resultant decreasing parasite load. This is the major effective malaria immunity possessed by adults who have lived most of their lives in malaria-endemic areas.^9^

In holoendemic malarious areas, people are repeatedly exposed to malaria infection from childhood and well into adulthood, with children manifesting more disease symptoms than adults.^9^,^3^,^10^ When a child is born in a holoendemic area, their risk of malaria infections is relatively low within the first six months of life through the protection from the passively-acquired immunity from placental transfer of maternal immunoglobin G.^9^,^10^,^11^ This passive immunity dwindles with age and the risk of disease and death increases.^9^ Through repeated exposure to sub-lethal infections, the child eventually develops active immunity from around two years.^9^ This immunity peaks around the age of puberty and gets sustained by subsequent repeated infections.^9^

Asymptomatic infection is the predominant presentation seen in patients with malaria immunity and is believed to confer some form of immunity through premunition.^9^,^10^ It is important to state categorically that this immunity is non-sterilizing and can be lost if there is no exposure to infection for 3–5 years.^9^,^10^,^12^

### Mechanism of Immune Response to Malaria

The immunity against malaria is quite complex but mainly targets the asexual forms of the parasite employing a timely relay between antibody and cell-mediated immunity.^9^,^12^ Malaria immunity characteristically begins with an innate immune response involving macrophages, dendritic cells and natural killer cells resulting in a sharp burst of pro-inflammatory cytokines that induce inflammation to restrict parasite growth.^9^,^12^ These pro-inflammatory cytokines must be regulated by anti-inflammatory cytokines because they can lead to severe malaria and death when unregulated.^9^,^12^ This is followed by activation of the CD4 cells with subsequent clonal expansion producing T_h_1 and T_h_2, resulting in cell-mediated and antibody-mediated immunities, respectively.^9^,^12^ T_h_2 CD4 cells activate B cells to produce antibodies, especially immunoglobin G, which play pivotal roles in the immunity against the parasite.^2^,^9^,^12^ Antibodies produced target free circulating merozoites and parasitized red blood cells (RBCs), preventing merozoites invasion of (RBCs) and the cytoadherence of parasitized RBCs on the endothelium^9^ This attachment of the antibodies to the merozoites and parasitized RBCs could result in any of the following: opsonization and subsequent phagocytosis, complement-mediated cell destruction or antibody-dependent cell-mediated destruction of parasitized RBCs.^9^,^12^ Immunoglobin G is the major antibody that triggers this cascade of immune reactions. In some malaria-endemic areas, high levels of circulating immunoglobin G have been linked to lower malaria risks.^12^,^13^,^14^ T_h_1 promotes cell-mediated immunity by aiding macrophages in the destruction of parasitized RBCs.^12^

Natural killer cells are activated in malaria infection, a finding seen among infected children in endemic areas.^12^,^15^,^16^ They are involved in the direct destruction of parasitized RBCs and the production of pro-inflammatory cytokines like interferon gamma.^12^,^17^ This occurs early in malaria infection, even before activation of CD4 cells.^12^ Interferon gamma has been linked with resistance to malaria infection, milder diseases in children and also prevention of reinfection in children within a year of mild infections.^17^,^18^,^19^ Interferon alpha and beta, collectively referred to as type 1 interferon also play an important role in malaria immunity by regulating the pro-inflammatory function of interferon gamma, thereby preventing an unbridled inflammatory response which can lead to severe disease.^20^

After the immune cells and their products (cytokines and antibodies) are produced during an immune response to malaria, they can linger for years as memory cells and as circulating cytokines and antibodies if sustained by repeated infections.^9^,^12^,^17^ Though natural killer cells have been considered part of innate immunity, and recent findings suggest a capacity to retain memory and elicit a memory-like response.^17^,^21^

### Immunity against Viruses

Like the immunity against malaria, immunity against viruses begins with innate immunity and chiefly involves dendritic cells, natural killer cells and pro-inflammatory cytokines, especially interferons.^22^,^23^ The interferons play a significant role in antiviral immunity. The name interferon derives from their ability to “interfere” with viral replication. The three major types, namely, types 1, 2 and 3 interferons, are all involved in antiviral immunity with type 2, also called interferon gamma, additionally playing a major role in immunity against malaria. While type 1 and type 3 are produced by infected host cells, interferon gamma is produced by immune cells like natural killer cells and the CD8- and CD4-bearing T-cells.^24^ Interferon gamma plays a complex and vital immune modulatory function regulating the actions of other interferons and cytokines and cellular immunity.^24^,^25^ Its activity in inhibiting the growth of both DNA and RNA viruses is evidenced by the decreased multiplication of the Varicella-Zoster virus within infected neurons resulting in the improved survival of the neurons.^26^ Interferon gamma also impedes the growth and multiplication of the Hepatitis C virus within infected hepatocytes.^27^ It decreases the multiplication of SARS-CoV, the coronavirus responsible SARS outbreak of 2003.^28^,^29^ Cytokine analyses of patients with SARS showed interferon gamma's presence at the early stages of the infection resulted in early resolution of the symptoms.^30^ Interferon therapy is consequently under evaluation as a treatment option for patients with COVID-19.^31^,^32^

Natural killer cells are the chief immune cells involved in cellular immunity to viruses.^33^,^34^ Their vital roles against viruses are highlighted in the heightened susceptibility of viral infections in persons with the rare congenital disorder of natural killer cell deficiency.^34^ Natural killer cells, very efficient in killing viruses without prior sensitization, have been noted to be potent in killing: Poxviruses, Papillomaviruses, Herpesviruses, including the Cytomegalovirus and Influenzavirus.^33^,^34^ The effect of natural killer cells on coronaviruses is underscored in SARS, where a decreased number of natural killer cells was linked to the severity of the disease.^35^ A similar observation has recently been noted in patients with COVID-19 with improved numbers of natural killer cells seen upon recovery after treatment with antiviral therapy, chloroquine, antibiotics and interferon.^36^ Natural killer cell therapy is under evaluation for the management of COVID-19.^37^

Antibodies are another product of the immune system that is very potent against viruses and efficiently prevent and resolve viral infections.^38^,^39^ Antibodies generally neutralize free circulating viruses, a potent way of preventing viral infection by preventing them from gaining access to target cells.^38^,^39^ Another way antibodies destroy viruses or virally infected cells are via Fc-mediated cell destruction using complement activation or antibody-mediated natural killer cell destruction.^38^,^39^ The importance of antibodies against viruses is underscored in patients with severe antibody deficiency seen in Burton's X-linked agammaglobulinemia (XLA). Severe viral infections in these patients resolve upon treatment with high doses of intravenous immunoglobin G.^39^ Immunoglobins G, A and M are crucial in antiviral immunity.^40^ Immunoglobin G appears pivotal in the recovery of persons with SARS and COVID-19.^41^,^42^ Production of immunoglobulins M and G were found to have started by the second week of infection in persons with SARS. As expected, immunoglobulin M levels rapidly began to fall after attaining their peak levels within the first few weeks, disappearing within months, while the immunoglobin G levels remained high for months after achieving peak levels^40^ Consequently, immunoglobulin G is thought to be the predominant antibody in the immune response to SARS-CoV, as is known to occur in other viral infections^41^ Intravenous immunoglobulin (IVIg), which is immunoglobulin G obtained from several healthy adults, is also under evaluation for use as treatment in combination with other antiviral agents in patients with COVID-19.^43^,^44^ Despite the fact that IVIg is most effective when obtained from persons who have recently recovered from COVID-19, IVIg from healthy persons without prior exposure to COVID-19 has been successfully employed in the treatment of persons with COVID-19.^44^

### Malaria and Viral Comorbidity

One of the ways to understand how acquired malaria immunity affects other pathogens like viruses are to understand what happens in coinfections with malaria.^45^ In malaria-endemic areas of the world, several viruses can infect humans, making it theoretically and clinically possible for viral diseases and malaria to co-exist in a single patient.^46^ How these diseases interact with each other is an expression of the rather complex alteration of the immune system they exert on the host, which affects their pathogeneses.^46^ For example, in a review of studies on malaria and HIV coinfection, pregnant women and children were found to bear the brunt of both infections due to the negative effects of each other, with malaria increasing HIV viral load and HIV increasing malaria parasitemia.^47^

A similar effect was seen in a study on malaria and Epstein-Barr virus coinfection, which established that malaria lowers antiviral immunity in some children resulting in uncontrolled growth of B lymphocytes leading to Burkitt's lymphoma. The Epstein-Barr virus can suppress anti-parasite immunity in some children resulting in noncerebral severe malaria.^48^ Malaria has also been known to antagonize Chikungunya virus in mice with coinfection.^49^ Studies of malaria and Ebola virus coinfection have yielded variable results with reports of increased survival in some patients and increased mortality risk in others.^50^,^51^,^52^

A scan of the available literature on malaria and respiratory virus coinfections in malaria endemic areas show that respiratory viruses are common in these areas and include: *Influenza virus, Rhinovirus*, human *Respiratory Syncytial Viruses* and coronaviruses, among others.^53^,^54^,^55^,^56^ These studies, however, were focused on the prevalence, clinical presentation and transmission dynamics of the viruses and did not consider the immune interactions and alterations occasioned by these coinfections. Malaria is considered a strong immunomodulator and is believed to hold potential benefit in the treatment of certain diseases.^57^

### Possible Relationships between Malaria-activated Innate Immunity and COVID-19

It is an established fact that malaria is a potent immunomodulator of adaptive immunity^9^ and has more recently been shown to also activate innate immunity, inducing immunological memory similar to adaptive immunity.^58^,^59^ This immunological memory of the innate immunity, capable of mounting swifter and more successful immune response against subsequent infections and also capable of providing cross protection, is called trained immunity.^58^,^59^ Trained immunity is a term used for the first time by Netea and colleagues in 2011,^58^ which explains the activation of innate immunity by certain vaccines like BCG^60^ and pathogens like malaria.^61^,^62^

The immune response against malaria involves innate immunity with natural killer cells, monocyte, macrophages, proinflamatory and anti-inflammatory cytokines as the major players.^9^,^15^,^17^ Thus, persons living in malaria endemic areas with malaria immunity acquired through repeated sub-patent infections, might possess trained immunity which can be beneficial in mounting successful immune response against other pathogens like SARS-CoV-2.^63^

Cross protection of trained immunity is highly likely because of the non-specific response of innate immunity, evident by BCG vaccination conferring protection against other pathogens aside *Mycobacterium tuberculosis.*^59^,^64^ Therefore, it is possible that those with malaria immunity possess trained immunity and have circulating primed innate cells like natural killer cells capable of mounting a prompt and successful response against SARS-CoV-2.^63^ Cellular actors involved in trained immunity; for example, the natural killer cells are swifter in the production cytokines effective against subsequent infections.^59^

Malaria activation of innate immunity produces not only trained immunity (hyperresponsiveness) but also tolerance (hyporesponsiveness).^61^ This tolerance ensures a minimum inflammatory response from innate immune cells like monocytes in persons with malaria immunity, curbing the harmful inflammatory effects responsible for severe malaria, common in those without malaria immunity^65^ This tolerance activated by malaria can also be cross-protective as subsequent unrelated infections might not be met with intense inflammatory response, thereby protecting against severe diseases.^66^ It has been speculated that this tolerance might be the reason for people living in malaria-endemic areas (and possessing malaria immunity) being protected against the severe inflammatory response of COVID-19, the hallmark of severe SARS-CoV-2 infection.^66^

Taken together, while malaria influences adaptive immunity,^9^,^11^ its impact is felt more with the activation of innate immunity resulting in both trained immunity and immune tolerance^61^,^62^, both of which are beneficial in protecting against severe malaria and may offer cross-protection against other diseases like COVID-19.^63^,^66^

### Possible Research Areas

To answer the question posed by this review, studies need to be conducted among patients with COVID-19 who live in malaria-endemic areas. Acquired malaria immunity must be assessed both in patients with COVID-19 and among healthy individuals. All immune system components characteristically seen in persons with acquired immunity for malaria, like natural killer cells, interferon (interferon gamma and type 1interferon) and immunoglobin G, should be evaluated among symptomatic and asymptomatic patients with COVID-19. The role of malaria-induced trained immunity and immune tolerance in patients with COVID-19 should also be investigated. It is important to note that not all persons living in malaria-endemic areas have acquired malaria immunity.^67^ Some might have lost the immunity or failed to develop it due to inadequate exposure to *Plasmodium* as the levels of malaria exposure even in endemic areas is variable.^68^

The exposure risk is higher for persons with lower socioeconomic status, especially those living in crowded or riparian communities, compared to those with higher socioeconomic status living in well-organized urban cities.^69^,^70^ Additionally, the coordinated activities of national malaria control programs have progressively reduced the risks of malaria infection in many populations in malaria-endemic areas.^67^ The consequences of this reduced risk on malaria immunity should be also be investigated.

## Conclusion

The COVID-19 pandemic presents a great opportunity for scientists, physicians and epidemiologists in malariaendemic areas to investigate the impact of endemic diseases like malaria on COVID -19 and future epidemics.

## References

[1] World Health Organization Malaria Factsheet.

[2] Barry A, Hansen D (2016). Naturally acquired immunity to malaria. Parasitology.

[3] Crompton PD, Moebius J, Portugal S, Waisberg M, Hart G, Garver LS (2014). Malaria immunity in man and mosquito: insights into unsolved mysteries of a deadly infectious disease. Annual Review of Immunology.

[4] Liu Y, Gayle AA, Wilder-Smith A, Rocklov J (2020). The reproductive number of COVID-19 is higher compared to SARS coronavirus. Journal of Travel Medicine.

[5] Gilbert M, Pullano G, Pinotti F, Valdano E, Poletto C, Boelle P (2020). Preparedness and vulnerability of African countries against importations of COVID-19 : a modelling study. The Lancet.

[6] Africa CDC Coronavirus Disease 2019 (COVID-19): Latest updates on the COVID-19 crisis from Africa CDC.

[7] Velavan TP, Meyer CG (2020). The COVID-19 epidemic. Tropical Medicine & International Health.

[8] Nachega JB, Seydi M, Zumla A (2020). The late arrival of COVID-19 in Africa-Mitigating pan-continental spread. Clin Infect Dis.

[9] Doolan DL, Doban C, Baird JK (2009). Acquired Immunity to Malaria. Clinical Microbiology Reviews.

[10] Keegan LT, Dushoff J (2013). Population-level effects of clinical immunity to malaria. BMC Infectious Diseases.

[11] Dobbs KR, Dent AE (2016). Plasmodium malaria and antimalarial antibodies in the first year of life. Parasitology.

[12] Tognon M (2013). Malaria and some polyomaviruses (SV40, BK, JC, and Merkel cell viruses). IARC Monographs on the Evaluation of Carcinogenic Risks to Humans.

[13] Israelsson E, Vafa M, Maiga B, Lysen A, Iriemenam NC, Dolo A (2008). Differences in Fcgamma receptor IIa genotypes and IgG subclass pattern of anti-malarial antibodies between sympatric ethnic groups in Mali. Malaria Journal.

[14] Nasr A, Iriemenam NC, Giha HA, Balogun HA, Anders RF, Troye-Blomberg M (2009). FcγRIIa (CD32) polymorphism and anti-malarial IgG subclass pattern among Fulani and sympatric ethnic groups living in eastern Sudan. Malaria Journal.

[15] Hansen DS, D'Ombrain MC, Schofield L (2007). The role of leukocytes bearing Natural Killer Complex receptors and Killer Immunoglobulin-like Receptors in the immunology of malaria. Current Opinion in Immunology.

[16] Ombrain MCD, Robinson LJ, Stanisic DI, Taraika J, Bernard N, Michon P (2008). Association of Early Interferon-Gamma Production with Immunity to Clinical Malaria : a Longitudinal Study among Papua New Guinean Children. Clinical Infectious Diseases.

[17] Wolf AS, Sherratt S, Riley EM (2017). NK cells: uncertain allies against malaria. Frontiers In Immunology.

[18] McCall MBB, Hopman J, Daou M, Maiga B, Dara V, Ploemen I (2010). Early Interferon gamma Response against *Plasmodium falciparum* Correlates with Interethnic Differences in Susceptibility to Parasitemia between Sympatric Fulani and Dogon in Mali. The Journal of Infectious Diseases.

[19] King T, Lamb T (2015). Interferon-γ: the Jekyll and Hyde of malaria. PLoS Pathogens.

[20] Mooney JP, Wassmer SC, Hafalla JC (2017). Type I interferon in malaria: a balancing act. Trends in Parasitology.

[21] Cerwenka A, Lanier LL (2016). Natural killer cell memory in infection, inflammation and cancer. Nature Reviews Immunology.

[22] Braciale TJ, Hahn YS (2013). Immunity to viruses. Immunological Reviews.

[23] Li S, Fu B, Meshram CD (2019). Innate Immune and Inflammatory Responses to Respiratory Viruses. Mediators of Inflammation.

[24] De Andrea M, Ravera R, Gioia D, Gariglio M, Landolfo S (2002). The interferon system: an overview. European Journal of Paediatric Neurology.

[25] Kak G, Raza M, Tiwari BK (2018). Interferon-gamma (IFN-γ): exploring its implications in infectious diseases. Biomolecular Concepts.

[26] Baird NL, Bowlin JL, Hotz TJ, Cohrs RJ, Gilden D (2015). Interferon gamma prolongs survival of varicella-zoster virus-infected human neurons in vitro. Journal of Virology.

[27] Grebely J, Feld JJ, Applegate T, Matthews GV, Hellard M, Sherker (2013). Plasma interferongamma-inducible protein-10 (IP-10) levels during acute hepatitis C virus infection. Hepatology.

[28] Sainz B, Mossel EC, Peters CJ, Garry RF (2004). Interferon-beta and interferon-gamma synergistically inhibit the replication of severe acute respiratory syndrome-associated coronavirus (SARS-CoV). Virology.

[29] Cinatl J, Morgenstern B, Bauer G, Chandra P, Rabenau H, Doerr HW (2003). Treatment of SARS with human interferons. The Lancet.

[30] Humar SK, Louie M, Loeb MB, Wilkinson JP, Greller LD, Somogyi R (2007). Interferon-Mediated Immunopathological. J Virol.

[31] Sallard E, Lescure FX, Yazdanpanah Y, Mentre F, Peiffer-Smadja N (2020). Type 1 interferons as a potential treatment against COVID-19. Antiviral Research.

[32] Mosaddeghi P, Negahdaripour M, Dehghani Z, Farahmandnejad M, Moghadami M, Nezafat N, Masoompour SM (2020). Therapeutic approaches for COVID-19 based on the dynamics of interferon-mediated immune responses. Current Signal Transduction Therapy.

[33] Brandstadter JD, Yang Y (2011). Natural killer cell responses to viral infection. Journal of Innate Immunity.

[34] van Erp EA, van Kampen MR, van Kasteren PB, de Wit J (2019). Viral Infection of Human Natural Killer Cells. Viruses.

[35] National Research Project for SARS, Beijing Group (2004). The involvement of natural killer cells in the pathogenesis of severe acute respiratory syndrome. American Journal of Clinical Pathology.

[36] Zheng M, Gao Y, Wang G, Song G, Liu S, Sun D, Xu Y, Tian Z (2020). Functional exhaustion of antiviral lymphocytes in COVID-19 patients. Cellular & Molecular Immunology.

[37] Xinjiang Medical University NK Cells Treatment for COVID-19.

[38] Sanna PP, Burton DR (2000). Role of antibodies in controlling viral disease: lessons from experiments of nature and gene knockouts. Journal of Virology.

[39] Law M, Hangartner L (2008). Antibodies against viruses: passive and active immunization. Current Opinion in Immunology.

[40] Woo PC, Lau SK, Wong BH, Chan KH, Chu CM, Tsoi HW (2004). Longitudinal profile of immunoglobulin G (IgG), IgM, and IgA antibodies against the severe acute respiratory syndrome (SARS) coronavirus nucleocapsid protein in patients with pneumonia due to the SARS coronavirus. Clin Diagn Lab Immunol.

[41] Li G, Chen X, Xu A (2003). Profile of specific antibodies to the SARS-associated coronavirus. New England Journal of Medicine.

[42] Jawhara S (2020). Could Intravenous immunoglobulin collected from recovered coronavirus patients protect against COVID-19 and strengthen the immune system of new patients?. International Journal of Molecular Sciences.

[43] Krause I, Wu R, Sherer Y, Patanik M, Peter JB, Shoenfeld Y (2002). In vitro antiviral and antibacterial activity of commercial intravenous immunoglobulin preparations-a potential role for adjuvant intravenous immunoglobulin therapy in infectious diseases. Transfusion Medicine.

[44] Cao W, Liu X, Bai T, Fan H, Hong K, Song H (2020). High-dose intravenous immunoglobulin as a therapeutic option for deteriorating patients with coronavirus disease 2019. Open Forum Infectious Diseases.

[45] Mabbott NA (2018). The influence of parasite infections on host immunity to coinfection with other pathogens. Frontiers in Immunology.

[46] McArdle AJ, Turkova A, Cunnington AJ (2018). When do coinfections matter?. Current Opinion in Infectious Diseases.

[47] Kwenti TE (2018). Malaria and HIV coinfection in sub-Saharan Africa: prevalence, impact, and treatment strategies. Research and Reports in Tropical Medicine.

[48] Matar CG, Anthony NR, O'Flaherty BM, Jacobs NT, Priyamvada L, Engwerda CR (2015). Gammaherpesvirus coinfection with malaria suppresses anti-parasitic humoral immunity. PLoS Pathogens.

[49] Shen SS, Qu XY, Zhang WZ, Li J, Lv Z (2019). Infection against infection: parasite antagonism against parasites, viruses and bacteria. Infectious Diseases of Poverty.

[50] Rosenke K, Adjemian J, Munster VJ, Marzi A, Falzarano D, Onyango CO (2016). *Plasmodium* parasitemia associated with increased survival in Ebola virus-infected patients. Clinical Infectious Diseases.

[51] Fettig N (2018). Ebola Virus and Malaria Coinfection: How Infection with One Pathogen May Protect Against Another. JEMI PEARLS.

[52] Waxman M, Aluisio AR, Rege S, Levine AC (2017). Characteristics and survival of patients with Ebola virus infection, malaria, or both in Sierra Leone : a retrospective cohort study. Lancet Infect Dis.

[53] Tine RC, Ndiaye LA, Niang MN, Kiori DE, Dia N, Gaye O (2018). Upper respiratory infections in a rural area with reduced malaria transmission in Senegal: a pathogens community study. BMC Infectious Diseases.

[54] Waitumbi JN, Kuypers J, Anyona SB, Koros JN, Polhemus ME, Gerlach J (2010). Outpatient upper respiratory tract viral infections in children with malaria symptoms in Western Kenya. The American Journal of Tropical Medicine and Hygiene.

[55] Namagambo B, Kayiwa JT, Byaruhanga T, Owor N, Nabukenya I, Bakamutumaho B (2014). Co-infection of malaria and influenza viruses in Uganda: A pilot study. International Journal of Infectious Diseases.

[56] Thompson MG, Breiman RF, Hamel MJ, Desai M, Emukule G, Khagayi S (2012). Influenza and malaria coinfection among young children in western Kenya, 2009–2011. The Journal of Infectious diseases.

[57] Frosch AE, John CC (2012). Immunomodulation in *Plasmodium falciparum* malaria: experiments in nature and their conflicting implications for potential therapeutic agents. Expert Review of Anti-infective Therapy.

[58] Netea MG, Quintin J, Van Der Meer JW (2011). Trained immunity: a memory for innate host defense. Cell Host & Microbe.

[59] Netea MG, Dominguez-Andres J, Barreiro LB, Chavakis T, Divangahi M, Fuchs E (2020). Defining trained immunity and its role in health and disease. Nature Reviews Immunology.

[60] Arts RJ, Moorlag SJ, Novakovic B, Li Y, Wang SY, Oosting M (2018). BCG vaccination protects against experimental viral infection in humans through the induction of cytokines associated with trained immunity. Cell Host & Microbe.

[61] Schrum JE, Crabtree JN, Dobbs KR, Kiritsy MC, Reed GW, Gazzinelli RT (2018). Cutting edge: *Plasmodium falciparum* induces trained innate immunity. The Journal of Immunology.

[62] Rochford R, Kazura J (2020). Introduction: Immunity to malaria. Immunological Reviews.

[63] Raham TF (2020). Malaria Endemicity Influence on COVID-19 Mortality: New Evidence Added to BCG and TB Prevalence. medRxiv.

[64] Sohrabi Y, Dos Santos JC, Dorenkamp M, Findeisen H, Godfrey R, Netea MG (2020). Trained immunity as a novel approach against COVID-19 with a focus on Bacillus Calmette-Guerin vaccine: mechanisms, challenges and perspectives. Clinical & Translational Immunology.

[65] Boutlis CS, Yeo TW, Anstey NM (2006). Malaria tolerance-for whom the cell tolls?. Trends in Parasitology.

[66] Guha R, Mathioudaki A, Doumbo S, Doumtabe D, Skinner J, Arora G (2020). *Plasmodium falciparum* malaria drives epigenetic reprogramming of human monocytes toward a regulatory phenotype. bioRxiv.

[67] Ghani AC, Sutherland CJ, Riley EM, Drakeley CJ, Griffin JT, Gosling RD (2009). Loss of population levels of immunity to malaria as a result of exposure-reducing interventions: consequences for interpretation of disease trends. PLoS One.

[68] Snow RW, Marsh K (2002). The consequences of reducing transmission of *Plasmodium falciparum* in Africa. Adv Parasitol.

[69] Degarege A, Fennie K, Degarege D, Chennupati S, Madhivanan P (2019). Improving socioeconomic status may reduce the burden of malaria in sub Saharan Africa: A systematic review and meta-analysis. PloS One.

[70] Catry T, Li Z, Roux E, Herbreteau V, Gurgel H, Mangeas M (2018). Wetlands and malaria in the Amazon: Guidelines for the use of synthetic aperture radar remote-sensing. International Journal of Environmental Research and Public Health.

